# Cardioprotective effects of prolyl hydroxylase inhibitors during reperfusion in myocardial infarction: A study using cardiosphere‐derived cells

**DOI:** 10.1113/EP093391

**Published:** 2026-03-13

**Authors:** Luca Salhöfer, Ulrike B. Hendgen‐Cotta, Joachim Fandrey, Timm Schreiber, Tristan Leu

**Affiliations:** ^1^ Institute of Physiology University Duisburg‐Essen Essen Germany; ^2^ Department of Cardiology and Vascular Medicine, West German Heart and Vascular Centre, Medical Faculty University of Duisburg‐Essen Essen Germany; ^3^ Institute of Physiology and Pathophysiology, and Centre for Biomedical Education and Research (ZBAF) University of Witten/Herdecke Witten Germany

**Keywords:** cardiosphere‐derived cells, HIF, ischaemia, myocardial infarction, PHI

## Abstract

Cardiosphere‐derived cells (CDCs) are a promising in vitro model for studying myocardial ischaemia–reperfusion (I–R) injury and testing potential therapeutic interventions. This study investigated the suitability of CDCs as a model for myocardial infarction (MI) and the effects of prolyl hydroxylase inhibitor (PHI) administration during reperfusion. CDCs were generated from neonatal mouse hearts and characterized by immunofluorescence, revealing a heterogeneous mixture of cardiomyocytes, smooth muscle cells, endothelial cells, and stem cells. The CDCs were subjected to oxygen–glucose deprivation (OGD) followed by reperfusion with or without the PHI dimethyloxalylglycine (DMOG). Cell viability, hypoxia‐inducible factor 1‐alpha (HIF‐1α) accumulation, and gene expression were analysed. The results showed that DMOG administration during reperfusion reduced lactate dehydrogenase release, indicating decreased cell death. HIF‐1α protein levels increased during OGD and were further stabilized by DMOG during reperfusion. The expression of HIF‐1α target genes, such as vascular endothelial growth factor (*Vegfa*), and genes involved in regeneration and cardiac function, including connective tissue growth factor (*Ctgf*), cyclin D2 (*Ccnd2*), and beta‐1 adrenergic receptor (*Adrb1*), was modulated by OGD and DMOG treatment. Comparisons with an in vivo mouse I–R injury model revealed similarities in gene expression patterns. In conclusion, CDCs serve as an effective in vitro model for studying I–R injury, closely resembling the in vivo situation. Furthermore, PHI administration during reperfusion reduces cell death and modulates the expression of genes involved in cardioprotection and regeneration, highlighting the potential of PHIs as a therapeutic strategy for I–R injury.

## Abstract (highlights)

- **What is the central question of this study?**
  Can Cardiosphere‐derived cells (CDCs) be used to model ischaemia–reperfusion (I–R) injury and what are the effects of administering a prolyl hydroxylase inhibitor (PHI) during reperfusion?
- **What is the main finding and its importance?**
  CDCs provide an effective in vitro model for studying I–R injury. The administration of PHI during reperfusion reduces cell death and modulates cardioprotective and regenerative gene expression. This highlights the therapeutic potential of PHIs in the treatment of myocardial infarction.

## 1 INTRODUCTION

In 1937, the first attempts to reduce myocardial infarct size were made (Gross et al., 1937), and in the following 80 years, many cardioprotective strategies have been devised and tested. However, only one treatment has withstood the test of time, early reperfusion of the occluded coronary artery. One approach that has attracted increasing interest is the use of drugs that activate endogenous adaptive programmes. The hypoxia‐inducible factor (HIF) system plays a central role in cellular adaptation to limited oxygen supply, and emerging evidence suggests that modification of the HIF axis could protect the myocardial tissue against ischaemic damage. HIF is a heterodimeric transcription factor composed of an oxygen‐regulated α‐subunit (HIF‐α) and constitutively expressed β‐subunit (HIF‐β) (Wang et al., 1995). The protein stability of HIF‐α subunits (HIF‐1α, HIF‐2α and HIF‐3α) is regulated by three oxygen‐ and iron‐dependent prolyl‐hydroxylase (PHD) enzymes (Epstein et al., 2001). In recent years, there has been tremendous progress in the development of small‐molecule inhibitors of PHD dioxygenases (PHI). The European Medicines Agency approved three molecules: roxadustat, vadadustat and daprodustat. Vadadustat and daprodustat were approved only for use in dialysis patients; however, the latter has since been withdrawn by the pharmaceutical company for commercial reasons (Locatelli et al., 2023). These compounds may also be useful in the treatment of I–R injury. It is widely accepted that HIF accumulation by hypoxic preconditioning, genetic approaches to reduce PHD activity, or PHIs is beneficial for I–R injury in animals, resulting in reduced infarct size and improved cardiac function (for review see Schreiber et al., 2019). Although many questions remain unanswered, in particular, whether the beneficial effects of PHI administration persist when the treatment occurs after an ischaemic event, such as in clinical settings.

Animal models are the gold standard for investigating myocardial I–R injury. Although it is difficult to adequately imitate the physiology of the human heart and working with large animals is very complex and expensive, many scientists resort to rodents. Compared to humans and pigs, however, they have a significantly higher heart rate and differ in electrophysiology, expression of myosin isoforms and metabolism (Gibbs, 2003; Hamlin & Altschuld, 2011; O'Hara & Rudy, 2012). The in vitro models used to date are primarily based on isolated cardiomyocytes. Although these models do not represent the complexity of the whole organ, they help analyse the effects of different interventions and other influencing factors, such as an ischaemic environment. In addition to hypoxic elements, factors such as hyperkalaemia, acidosis, nutrient deficiency and accumulation of metabolic products should also be considered (Kalogeris et al., 2012). To better study the organ in its entirety, cardiosphere‐derived cells (CDCs) may be a useful model for I–R injury research. CDCs can be obtained from the culture outgrowth of animal or human myocardial tissue and consist of a heterogeneous mixture of different cell types, including mesenchymal stem cells (MSCs), c‐kit^+^ cells, endothelial cells and other less‐well‐defined populations. They also show the potential to differentiate into the three most important cell lines in the heart: cardiomyocytes, smooth muscle cells and endothelial cells (Carr et al., 2011). CDCs are mainly used as a source of myocardial progenitor cells that can be transplanted into test animals or human patients. However, to our knowledge, CDCs have never been used as I–R injury models. In this study, we examined whether CDCs are a suitable model for the study of myocardial infarction (MI) and, in particular, whether the administration of PHIs during reperfusion is beneficial for cardiac cells.

## 2 METHODS

### 2.1 Ethical approval

All animal procedures complied with the policies of *Experimental Physiology* regarding animal experimentation and were conducted in accordance with German animal welfare law and institutional guidelines. All experiments were approved by the State Agency for Nature, Environment and Consumer Protection North Rhine‐Westphalia (in vitro: 84‐02.04.2016.A173; in vivo: G1498/15 AZ84‐02.04.2014.A144). Wild‐type C57BL/6J mice were used for all in vitro and in vivo experiments. Animals were housed under standard conditions with complete pelleted feed and drinking water provided ad libitum, and all mice exhibited a normal physiological condition and breeding behaviour. Deep anaesthesia for experimental procedures was induced using isoflurane (1.2–2 vol.%) according to approved protocols. Mice used for in vivo experiments were euthanized under deep anaesthesia (4–5 vol.%) by heart excision, and animals used for primary cell isolation (postembryonic stage P2–P4) were euthanized by decapitation before heart collection.

### 2.2 Cell culture

The generation of the CDCs was adapted according to Tan et al. (2011), and they were transferred to neonatal mice as follows. The heart of the neonatal mouse was removed and placed in a Petri dish containing phosphate‐buffered saline (PBS). The heart was divided into smaller fragments and washed in PBS to ensure all blood residues were removed. The tissue fragments were then transferred to a Petri dish containing trypsin for 3 min, after which they were placed in a dish containing complete explant medium (CEM; IMDM (Thermo Fisher Scientific, Waltham, MA, USA), supplemented with 20% fetal bovine serum (FBS; Biochrom AG, Berlin, Germany), 100 U/ml penicillin and 100 µg/ml streptomycin (both Merck KGaA, Darmstadt, Germany), and 0.1 mM β‐mercaptoethanol (Merck)) and divided further until the fragments were approximately 1.5 mm^3^. The tissue fragments were then transferred to a 60 mm Petri dish coated with fibronectin and incubated at 37°C with 5% CO_2_ for 24 h, after which 0.5 mL of CEM was added to the explant culture to protect the cells from dehydration. To harvest the explant‐derived cells (EDCs), the medium was removed from the explant culture, after which the cells were washed twice with PBS. The adherent cells were dissociated using trypsin and transferred to a centrifuge tube. The EDCs were then centrifuged for 5 min at 800 *g*. The supernatant was discarded, and the cells were resuspended in 1 mL of cardiosphere growth medium (CGM; 60% DMEM/F12 with 30% IMDM (both Thermo Fisher Scientific), supplemented with 7% FBS (Biochrom), 100 U/ml penicillin and 100 µg/ml streptomycin (both Merck), 0.1 mM β‐mercaptoethanol (Merck), 2% v/v B27 supplement (Thermo Fisher Scientific), 25 ng/ml cardiotrophin, 10 ng/ml epidermal growth factor (EGF), 20 ng/ml basic fibroblast growth factor (bFGF), and 5 U/ml thrombin (all PreproTech GmbH, Hamburg, Germany). One hundred thousand cells were added to a plate coated with 160 µg/ml poly‐d‐lysine (PDL; Merck) and incubated at 37°C. Cardiospheres developed after 2–3 days and increased in size over time. To obtain CDCs, the cardiospheres were removed from the plate by flushing with PBS. The semi‐adherent cardiospheres were dissociated by continuous pipetting with a 1 mL pipette tip, after which they were collected in a centrifuge tube. After centrifugation for 5 min at 800 *g*, the supernatant was discarded and the cell pellet was resuspended in 7 mL CEM. The cell suspension was then transferred to a fibronectin‐coated culture bottle and incubated at 37°C.

### 2.3 Oxygen–glucose deprivation

To simulate a MI, the CDCs were placed in a hypoxia chamber for up to 8 h in a glucose‐free medium with 0.2% oxygen. This process is called oxygen–glucose deprivation (OGD). RNA and protein were isolated after 1, 2, 4, 6 and 8 h of OGD. After each of these time points, the cells were reperfused with 1 mM DMOG‐transposed CEM in an incubator at 20.9% O_2_. Following each treatment, RNA and protein samples were collected. To determine the concentration of lactate dehydrogenase (LDH), the medium was collected and measured using the Pierce LDH Cytotoxicity Assay Kit according to the manufacturer's instructions (Thermo Fisher Scientific).

### 2.4 in vivo I–R injury model

The in vivo experiments were performed by the CardioScienceLabs at the Department of Cardiology and Vascular Medicine of the University Hospital Essen according to their published protocol (Totzeck et al., 2016). Briefly, animals were anaesthetized with ketamine (100 mg/kg, intraperitoneally, i.p.) and xylazine (10 mg/kg, i.p.), orally intubated and ventilated throughout the surgery, with 0.8 L/min compressed air and 0.2 L/min O_2_ at a tidal volume of 250 µL/stroke and a breathing frequency of 140 strokes/min. Deep anaesthesia was maintained with isoflurane (1.2–2 vol.), administered via the ventilation gas mixture. Following induction of deep anaesthesia, a lateral thoracotomy was made to expose the heart. Transient left coronary artery occlusion was performed for 30 min, followed by 60 min of reperfusion in deep anaesthesia. Mice were treated with 0.1 mg/kg buprenorphine subcutaneously after operation. At the end of the reperfusion period, the heart was excised in deep anaesthesia (4–5 vol.%), rinsed to remove residual blood, and immersion‐fixed in formalin for subsequent histological analysis.

### 2.5 Immunohistochemistry

For immunofluorescence staining, the cells were fixed with 4% paraformaldehyde. The cells were then permeabilized for 10 mins in 0.3% PBS‐T and the non‐specific binding sites were blocked with 10% goat serum/PBS‐T for 60 min. The antibodies were then diluted in 0.1% PBS‐T. The following primary antibodies were used: anti‐actin, α‐sarcomeric (cat. no. A2172), anti‐Actin, α‐smooth muscle (cat. no. A2547), anti‐von Willebrand factor (cat. no. AB7356) and anti‐Oct3/4 (cat. no. O8389, all from Merck). The appropriate anti‐mouse and anti‐rabbit Alexa 488 and Alexa 468 fluorochrome antibodies (Thermo Fisher Scientific) were then used. To visualize the nuclei further, the cells were covered with Mowiol containing 4′,6‐diamidino‐2‐phenylindole (DAPI). Quantification was performed from three randomly selected fields per independent isolation, and the proportion of marker‐positive cells was calculated relative to the total number of nuclei.

For tissue analysis, the organs were fixed and drained. The dehydrated tissue samples were then embedded in paraffin wax and cut into 4 µm‐thick sections. Further implementation followed the (CSA II kit‐Dako Agilent, Santa Clara, USA) instructions (CSA II Biotin‐Free Tyramide Signal Amplification System, Edition 12/18). The tissue was stained with a primary antibody that targeted HIF‐1α (Cayman Chemical Co., Midland, MI, USA, cat. no. 10006421).

### 2.6 Western blot

Western blot analysis was performed as previously described (Wobben et al., 2013), using 20 µg of protein lysate per sample. An antibody targeting HIF‐1α (Cayman Chemical, cat. no. 10006421) was used. Visualization was induced using a goat anti‐rabbit immunoglobulin/horseradish peroxidase (Merck, cat. no. ab97051) antibody.

### 2.7 PCR

Total RNA was isolated from CDCs using a NucleoSpin RNA kit (Macherey‐Nagel GmbH & Co. KG, Düren, Germany).

cDNA was synthesized using M‐MLV reverse transcriptase (Promega GmbH, Walldorf, Germany) and qPCR analysis was performed using a Biozym Blue S'Green qPCR kit (Biozym Scientific GmbH, Oldendorf, Germany) on a Bio‐Rad CFX96 real‐time system (Bio‐Rad Laboratories GmbH, Feldkirchen, Germany).

The following primer pairs were used: *Ldha*: 5′‐GTCTCCAGCAAAGACTACTGT‐3′ forward and 5′‐ GACTGTACTTGACAATGTTGGGA‐3′ reverse; *Ccnd2*: 5′‐CGCACAGAGCGATGAAGGT‐3′ forward and 5′‐GAGTGGGAACTGGTAGTGTTG‐3′ reverse; *Cdk2*: 5′‐CCTGCTTATCAATGCAGAGGG‐3′ forward and 5′‐GTGCTGGGTACACACTAGGTG‐3′ reverse; *Ctgf*: 5′‐ATCCAGGCAAGTGCATTGGTA‐3′ forward and 5′‐GGGCCTCTTCTGCGATTTC‐3′ reverse; *Gja1*: 5′‐GAGAGATGGGGAAGGACTTGT‐3′ forward and 5′‐ACAGCGGTTGAGTCAGCTTG‐3′ reverse; *Adrb1*: 5′‐ACACACAGCACATCTACCGAA‐3′ forward and 5′‐CTCATCGTGGTGGGTAACGTG‐3′ reverse; *Vegfa*: 5′‐ACTGGACCCTGGCTTTACTG‐3′ forward and 5′‐ACTTGATCACTTCATGGGACTTCT‐3′ reverse; *Phd2* 5′‐TTGTTACCCAGGCAACGGAAC‐3′ forward and 5′‐CCTTGGCGTCCCAGTCTTT‐3′ reverse; *Phd3* 5′‐AGGCAATGGTGGCTTGCTATC‐3′ forward and 5′‐GCGTCCCAATTCTTATTCAGGT‐3′ reverse; *Shh* 5′‐ TTCGGAGTTTCTTGTGATCTTCC‐3′ forward and 5′‐ AAAGCTGACCCCTTTAGCCTA‐3′ reverse; and *Pdgfa* 5′‐ TGCTGTGGATCTGACTTCGAG‐3′ forward and 5′‐ GAGGAAGCCGAGATACCCC‐3′ reverse.

### 2.8 Data analysis

Immunofluorescence staining was visualized using an Axiovert 200M (Zeiss Jena, Germany) fluorescence microscope and recorded with a DS‐Ri1 camera system and NIS Elements F.3.0 imaging software. The ‘ImageJ’ program from the National Institutes of Health (Bethesda, MD, USA) was used to superimpose the colour channels. Immunohistochemical staining was digitized using the Aperio ScanScope and analysed using the Aperio ImageScope programme.

### 2.9 Statistics

The results were evaluated using Prism 9 software (GraphPad Software, Boston, MA, USA). in vitro data were analysed using a two‐way analysis of variance (ANOVA) with *time* and *treatment* (OGD only, OGD + 1 h reperfusion, OGD + 1 h reperfusion + DMOG) as fixed factors to evaluate their main effects and interaction. When significant effects were detected, *post hoc* multiple comparisons of means were performed using Tukey's honestly significant difference (HSD) test. A two‐sided, unpaired, parametric Student's *t*‐test was performed to determine statistical significance for in vivo data. Results are presented as means ± SD, and statistical significance was set at *P* < 0.05*, *P* < 0.01**, *P* < 0.001***. Exact *P*‐values for in vitro experiments can be found in Table A1 in the Appendix. Each treatment and analysis from an independent isolation was considered an *n*.

## 3 RESULTS

### 3.1 Composition of CDCs

**FIGURE 1:**
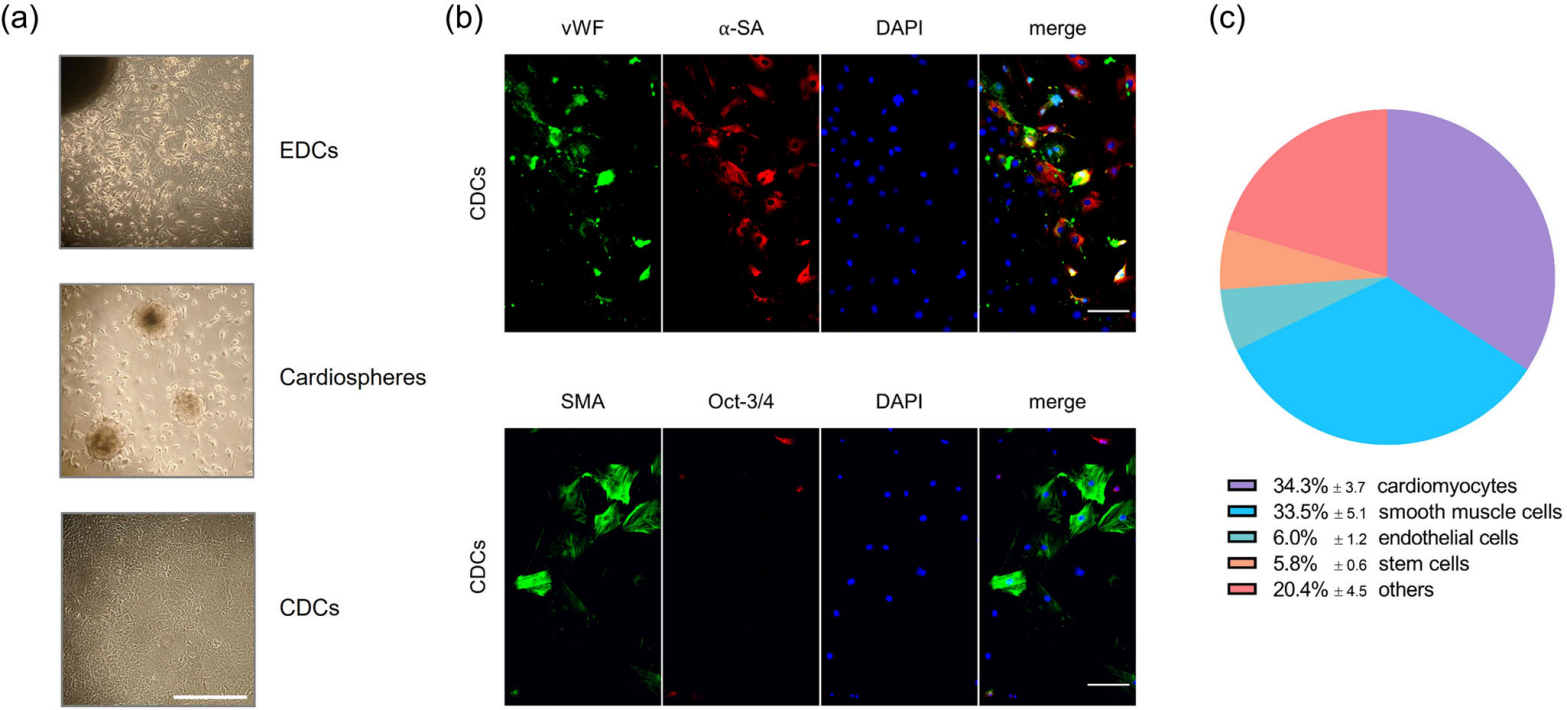
Generation and characterization of cardiosphere‐derived cells (CDCs). (a) Schematic illustration of the isolation, formation and expansion of CDCs from neonatal mouse hearts. Explant‐derived cells (EDCs) migrated out from tissue explants under culture, formed cardiospheres, and subsequently developed into an adherent population of cardiosphere‐derived cells (CDCs). Images acquired by transmission light microscopy. Scale bar: 100 µm. (b) Immunofluorescence characterization of CDCs, demonstrating cellular heterogeneity within culture. Endothelial cells were identified using vWF (green), smooth muscle cells with SMA (green), cardiomyocytes with α‐SA (red), and stem cells with Oct‐3/4 (red). Nuclei were counterstained with DAPI (blue). Representative staining shows predominant populations of cardiomyocytes and smooth muscle cells, with fewer endothelial and stem cells. Scale bar: 20 µm. (c) Quantification of cellular composition within CDC cultures. Data are presented as means ± SD. *n* = 3 (three random fields per independent isolations).

CDCs are mixtures of different cell types (Hsiao et al., 2014). To determine the composition of our model system, the CDCs were generated in three steps. The first step was harvesting EDCs from the hearts of neonatal mice (Figure 1a). These cells were induced to form cardiospheres using bFGF, EGF and thrombin and were then separated into CDCs. The presence of different cell types was investigated by immunofluorescence. Cardiomyocytes were detected by using antibodies against α‐sarcomeric actin (α‐SA). The presence of endothelial cells, smooth muscle cells and stem cells was also tested using antibodies against von Willebrand factor (vWF), smooth muscle actin, and octamer‐binding transcription factor 3/4 (Oct 3/4) (Figure 1b). In addition to qualitative detection, the proportions of different cell populations were determined by cell counting. In our cultured CDCs, muscle cells comprised the largest proportion: 34.3 ± 3.7% of all cells studied were cardiomyocytes, followed by 33.5 ± 5.1% of smooth muscle cells. Endothelial cells were present in much smaller numbers (6 ± 1.2%), as were stem cells at 5.8 ± 0.6% (Figure 1c, *n* = 3 independent isolations). Cells remaining unidentified amounted to 20.4 ± 4.5%.

### 3.2 HIF‐1α accumulation

**FIGURE 2:**
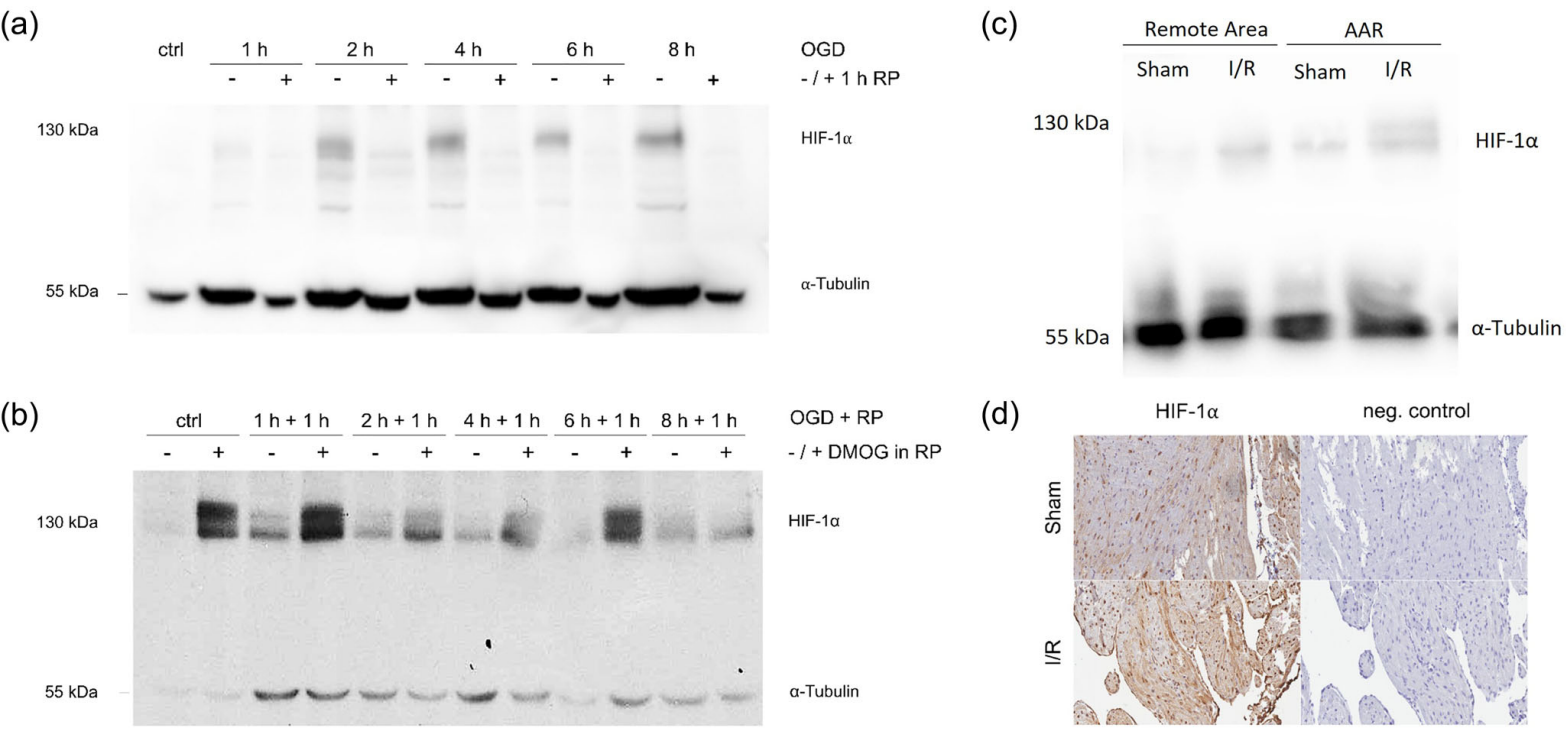
HIF‐1α protein regulation under ischaemic stress in vitro and in vivo. (a–b) Western blot analysis of HIF‐1α in CDCs subjected to oxygen–glucose deprivation (OGD), subsequent reperfusion (RP), and RP with DMOG treatment. α‐Tubulin served as the loading control. OGD increased HIF‐1α protein levels at all time points compared with control, while RP led to a reduction after 1 h. (a) HIF‐1α stabilization was enhanced when DMOG was present during RP. (b, c) in vivo western blot detection of HIF‐1α following LCA‐induced myocardial infarction or sham operation, comparing the area at risk (AAR) with remote myocardium. HIF‐1α was elevated in tissue post‐ligation relative to sham controls. (d) Immunohistochemical staining of HIF‐1α in cardiac tissue following ischaemia–reperfusion (I/R) or sham surgery, demonstrating increased immunoreactivity post‐infarction. *n* = 3 (Blots per independent isolations). Scale bar: 200 µm.

To assess whether the cells reacted to OGD by accumulating HIF‐1α protein, we tested HIF‐1α accumulation using Western blot analysis. An increase in HIF‐1α protein was observed after any period of OGD (Figure 2a, *n* = 3 independent isolations). As the OGD progressed, the HIF‐1α signal increased further. Following reperfusion (RP), a lower HIF‐1α signal was detected. To test the function of DMOG as a PHD inhibitor (PHI) and thus as a stabilizer of the HIF‐1α subunit in CDCs, the accumulation of HIF‐1α after RP with and without DMOG was examined. An increase in HIF‐1α protein level was observed in both the control group and after reperfusion when DMOG was added to the culture medium (Figure 2b, *n* = 3 independent isolations). In an in vivo mouse myocardial I–R model, HIF‐1α was stabilized in both the remote area and the area at risk (AAR) after ligation of the left coronary artery (LCA) followed by reperfusion and sham surgery (Figure 2c, *n* = 3 independent isolations). The AAR corresponds to the tissue affected by ischaemia, while the remote area comprises non‐ischaemic tissue. As HIF‐1α could be detected in the Western blot after both I–R and the sham‐OP, immunohistochemical (IHC) staining of HIF‐1α was performed for confirmation. Immunohistochemistry also allowed conclusions to be drawn regarding the localization of the protein. The staining indicated that HIF‐1α was stabilized in the tissue sections after sham surgery and I–R (Figure 2d, *n* = 3 independent isolations). However, the staining appeared to be stronger after I–R.

### 3.3 Viability with PHI

**FIGURE 3:**
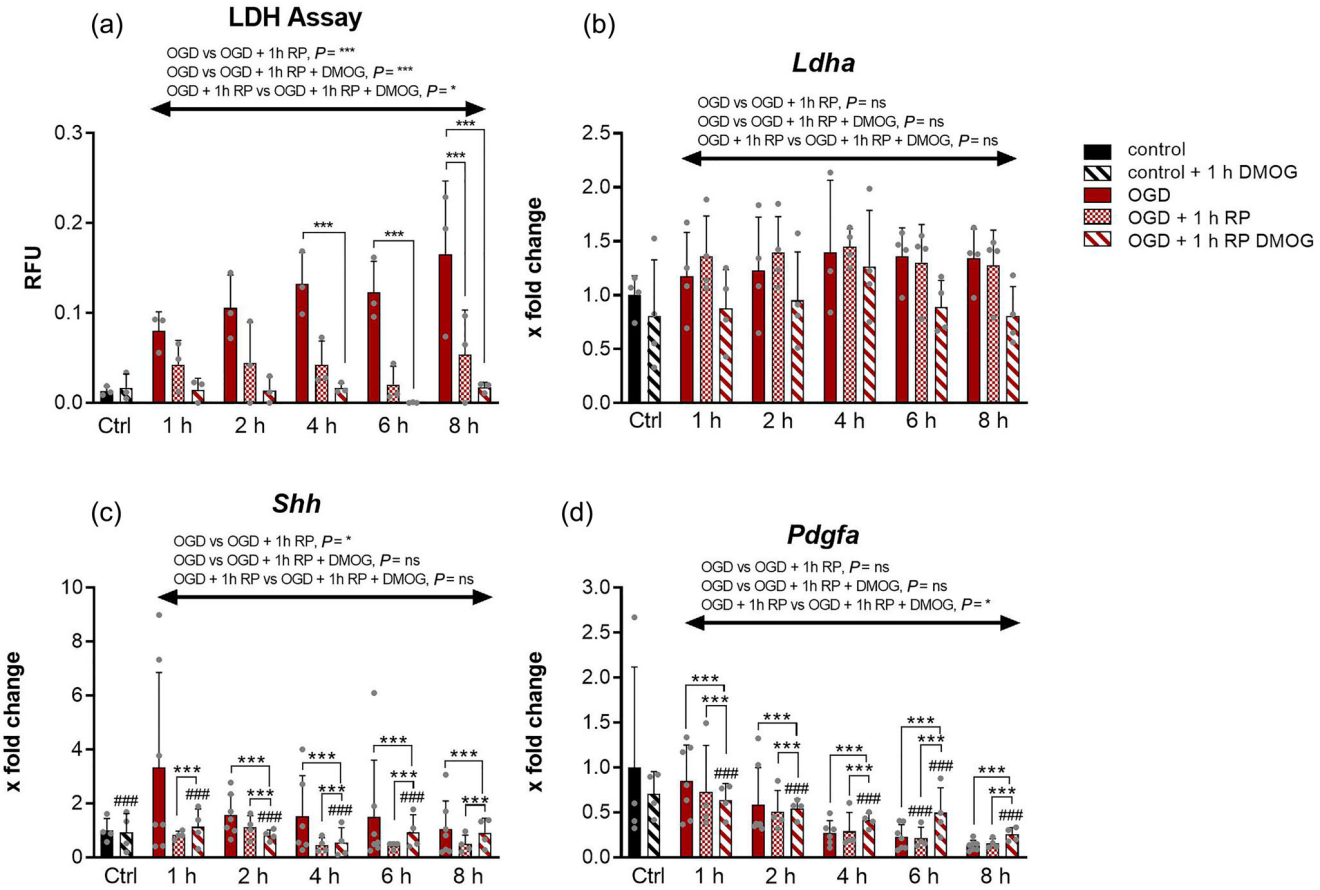
Metabolic injury and gene expression dynamics in cardiospheres subjected to ischaemic stress in vitro. (a) LDH release assay performed on cardiospheres exposed to oxygen–glucose deprivation (OGD), OGD + reperfusion, or OGD + reperfusion with DMOG treatment, assessed across 1, 2, 4, 6 and 8 h. *n* = 3 (measurement per independent isolations. (b–d) in vitro RT‐qPCR analysis of *Ldha*, *Shh* and *Pdgfa* expression in the same treatment groups and time points. Expression values were normalized to untreated controls. Experimental replicates: OGD, *n* = 4–7; OGD + reperfusion, *n* = 4; OGD + reperfusion + DMOG, *n* = 4 (analyses per independent isolation). Statistical analysis was conducted using two‐way ANOVA for the effects of time, treatment and their interaction, with an additional mean (treatment/column) comparison across time points. Data are presented as means ± SD. #Significant difference vs. control group; *significant difference between experimental groups. Significance levels: *P* < 0.05, *P* < 0.01, *P* < 0.001.

OGD poses a significant challenge for CDCs. To assess cell death and survival, we investigated the release of LDH into the cell culture medium. Measurement of LDH release showed an increase over the course of the experiment compared with the untreated control at 20.9% O_2_ (Figure 3a, *n* = 3 independent isolations). Following 1 h of reperfusion, there was no significant increase in LDH release compared to that in the control. Compared to LDH release at the respective OGD time points, lower LDH release was observed in all cases. To determine whether the addition of a prolyl hydroxylase inhibitor (PHI) had an effect in the short‐term during reperfusion, the influence of the PHI DMOG on reperfusion after previous OGD was investigated in vitro. After 1 h of RP with DMOG, a significant change in LDH release compared with the control was observed after 4, 6 and 8 h of OGD. However, there was a significantly lower release compared to RP without DMOG across all time points (Figure 3a). To rule out the possibility that the increased LDH release was due to increased *Ldha* expression, we performed qPCR analyses. There was no significant change in *Ldha* expression during any of the OGD periods or the subsequent RP. There was also no significant difference between the treatment groups over the complete course of the experiment (Figure 3b, OGD, *n* = 4–7; OGD + reperfusion, *n* = 4; OGD + reperfusion + DMOG, *n* = 4). To assess whether pro‐survival signalling pathways contribute to the protective effects of DMOG, we examined the expression of *Sonic hedgehog* (*Shh*) and *Pdgfa* following OGD and reperfusion. OGD alone did not significantly affect *Shh* transcript levels at any time point examined (Figure 3c, OGD, *n* = 4–7; OGD + reperfusion, *n* = 4; OGD + reperfusion + DMOG, *n* = 4). DMOG treatment during reperfusion resulted in an increase in *Shh* expression at 4, 6 and 8 h of reperfusion compared to reperfused cells without DMOG. Over the entire course of the experiment, reperfusion injury led to a significant reduction in *Shh* expression, whereas no significant reduction was observed in the presence of DMOG. In the case of *Pdgfa*, transcript levels progressively decreased over time after OGD (Figure 3d, OGD, *n* = 4–7; OGD + reperfusion, *n* = 4; OGD + reperfusion + DMOG, *n* = 4). However, DMOG administration during reperfusion attenuated this decline and resulted in elevated *Pdgfa* expression at later reperfusion time points compared with reperfusion alone. Across all time points, DMOG administration significantly increased *Pdgfa* expression compared to OGD alone. These DMOG‐associated changes in *Shh* and *Pdgfa* expression temporally align with the modest reductions in LDH release, supporting the notion that DMOG engages pro‐survival transcriptional programmes that may contribute to cytoprotection during reperfusion.

### 3.4 Gene expression of HIF‐1α targets and regulating enzymes

**FIGURE 4:**
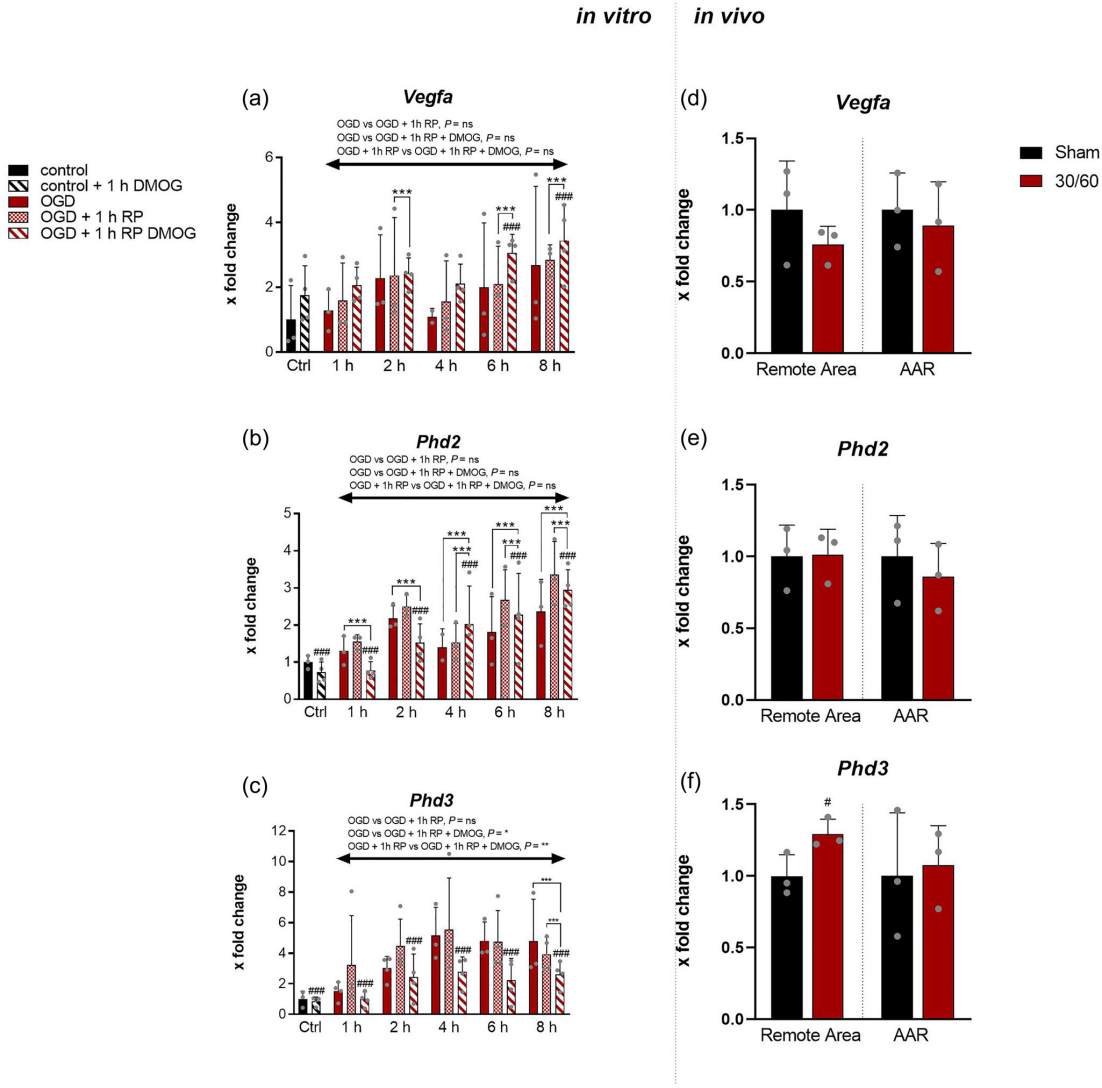
*Vegfa*, *Phd2*, and *Phd3* expression in vitro and in vivo following ischaemic stress. (a–c) in vitro real‐time PCR analysis of *Vegfa*, *Phd2* and *Phd3* expression in cardiospheres subjected to (i) oxygen–glucose deprivation (OGD), (ii) OGD + reperfusion, or (iii) OGD + reperfusion with DMOG treatment. Gene expression was quantified at 1, 2, 4, 6 and 8 h and normalized to control. Experimental replicates: OGD, *n* = 3; OGD + reperfusion, *n* = 3; OGD + reperfusion + DMOG, *n* = 4 (analyses per independent isolation). Statistical evaluation was performed by two‐way ANOVA for the effects of time, treatment, and their interaction, including a separate treatment (mean column) analysis across pooled time points. (d–f) in vivo real‐time PCR analysis of *Vegfa*, *Phd2*, and *Phd3* expression in myocardial tissue following LAD‐induced myocardial infarction or sham surgery. Samples were collected from the area at risk (AAR) and the remote myocardium across three independent experiments (*n* = 3). Group differences were assessed using an unpaired *t*‐test. Data are presented as means ± SD. #Significant difference vs. respective control group; *significant difference between experimental groups. Significance thresholds: *P* < 0.05, *P* < 0.01, *P* < 0.001.

Vascular endothelial growth factor (VEGF) induces the migration and proliferation of vascular endothelial cells, and is therefore essential for both physiological and pathological angiogenesis. DMOG administration significantly elevated *Vegfa* expression at later time points compared to reperfusion without DMOG (Figure 4a, OGD, *n* = 3; OGD + reperfusion, *n* = 3; OGD + reperfusion + DMOG, *n* = 4), but no significant intergroup differences were detected. PHDs lead to degradation of the HIF‐α subunit and act as oxygen sensors in all human cells. *Phd2* expression increased during the course of the experimental process (Figure 4b, OGD, *n* = 3; OGD + reperfusion, *n* = 3; OGD + reperfusion + DMOG, *n* = 4). After 6 and 8 h, the expression in *Phd2* in RP with DMOG were significantly lower compared to RP without DMOG. A similar difference in expression was observed at all time points analysed for *Phd3* expression (Figure 4c, OGD, *n* = 3; OGD + reperfusion, *n* = 3; OGD + reperfusion + DMOG, *n* = 4). Following OGD and subsequent RP without DMOG, the expression gradually increased up to 4 h. Interestingly, the administration of DMOG during reperfusion decreased *Phd3* expression compared to reperfusion without DMOG across all time points. Following LCA occlusion and reperfusion in vivo, *Vegf* expression in the remote area and AAR was reduced compared with that in the control (Figure 4d, *n* = 3 independent operations). *Phd2* and *Phd3* showed different effects; *Phd2* was downregulated after I–R in the AAR, while Phd3 was expressed at significantly higher levels in the remote area than in the control group (Figure 4e, f, *n* = 3 independent operations).

### 3.5 Expression of genes involved in regeneration

**FIGURE 5:**
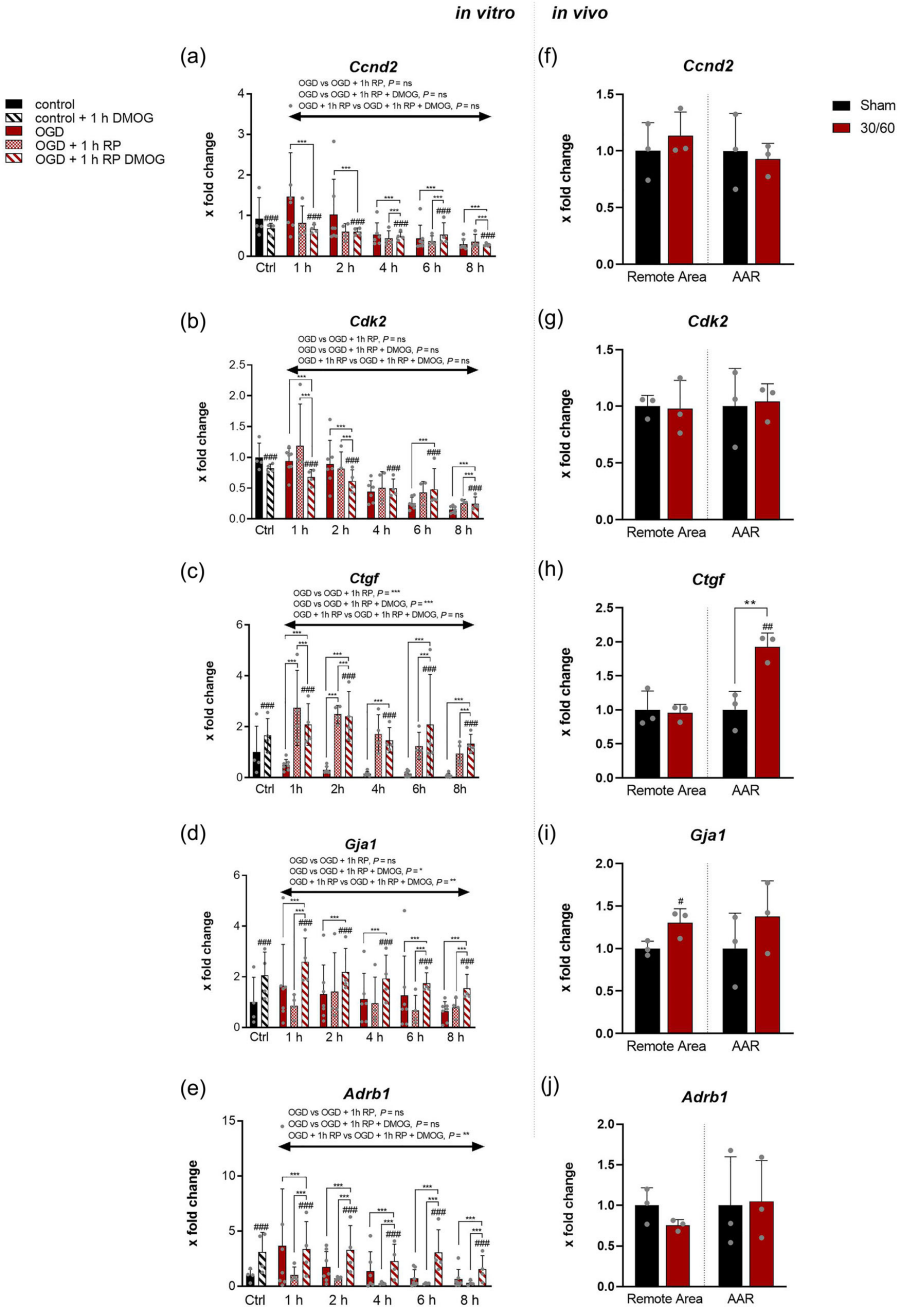
Expression of *Ccnd2*, *Cdk2*, *Ctgf*, *Gja1* and *Adrb1 in vitro* and in vivo following ischaemic stress. (a–e) in vitro real‐time PCR analysis of *Ccnd2*, *Cdk2*, *Ctgf*, *Gja1* and *Adrb1* expression in cardiospheres subjected to (i) oxygen–glucose deprivation (OGD), (ii) OGD + reperfusion, or (iii) OGD + reperfusion with DMOG treatment. Gene expression was assessed at 1, 2, 4, 6 and 8 h and normalized to untreated controls. Experimental replicates: OGD, *n* = 7; OGD + reperfusion, *n* = 4; OGD + reperfusion + DMOG, *n* = 4 (analyses per independent isolation). Statistical analysis was performed using two‐way ANOVA to evaluate effects of time, treatment, and their interaction, including a separate mean (treatment/column) effect across all time points. (f–j) in vivo real‐time PCR analysis of *Ccnd2*, *Cdk2*, *Ctgf*, *Gja1* and *Adrb1* expression in myocardial tissue following LAD‐induced myocardial infarction or sham operation. Tissue was harvested from the area at risk (AAR) and the remote myocardium from three independent experiments (*n* = 3). Statistical differences between groups were determined by unpaired *t*‐test. Data are presented as means ± SD. #Significant difference vs. control group; *significant difference between treatment groups. Significance levels: *P* < 0.05, *P* < 0.01, *P* < 0.001.

The cell cycle and its regulation are important factors in acute and chronic responses to I–R in the heart. Cyclin D2 (Ccnd2), a regulator of cyclin‐dependent kinases, is specifically involved in the transition from the G1 phase to the S phase. In our experiment, the *Ccnd2* gene, which encodes Ccnd2, showed a tendency for gradual reduction in expression (Figure 5a, OGD, *n* = 7; OGD + reperfusion, *n* = 4; OGD + reperfusion + DMOG, *n* = 4). However, no significant changes across all time points were observed. Cyclin‐dependent kinase 2 (Cdk2) belongs to the serine/threonine protein kinase group and regulates the transition of cells from G1 to S phase of the cell cycle. As with *Ccnd2*, there were no significant changes in *Cdk2* expression over the course of the experiment (Figure 5b, OGD, *n* = 7; OGD + reperfusion, *n* = 4; OGD + reperfusion + DMOG, *n* = 4). In the in vivo analysis, no differences in *Ccnd2* and *Cdk2* expression were detected during the experimental procedure. Connective tissue growth factor (Ctgf) is a matrix‐like protein that orchestrates processes such as cell division, differentiation, and angiogenesis under physiological conditions. Following OGD, a reduction in *Ctgf* expression was observed at all the time points (Figure 5c, OGD, *n* = 7; OGD + reperfusion, *n* = 4; OGD + reperfusion + DMOG, *n* = 4). *Ctgf* expression was significantly higher after RP, with and without DMOG, than after OGD across all time points. Compared to the control group, a significantly increased expression was observed at all time points of OGD plus subsequent RP with DMOG. In the mouse in vivo myocardial I–R model, *Ctgf* expression in the AAR following I–R was significantly higher than that in the remote area and the AAR after sham surgery (Figure 5h, *n* = 3 independent operations).

### 3.6 Expression of functional genes

Physiologically, Connexin 43, a gap junction protein and product of the *Gja1* gene, enables smooth tuning of cardiomyocytes via ion currents. During the course of the experiment, there was no significant change in *Gja1* expression after OGD or RP without DMOG (Figure 5d, OGD, *n* = 7; OGD + reperfusion, *n* = 4; OGD + reperfusion + DMOG, *n* = 4). However, after RP with DMOG, a significant increase in *Gja1* expression was observed at all time points of OGD, as well as across the entire experiment. According to in vivo I–R, *Gja1* expression in both the remote area and AAR was compared to the control group (Figure 5i, *n* = 3 independent operations). In the remote area, the increase compared to sham surgery was significant. As catecholamine receptors, adrenoceptors play a crucial role in vegetative adaptation processes. In the context of myocardial ischaemia, maintaining b1‐receptors is important for preventing heart failure (White et al., 2000). In CDCs, the expression of the *Adrb1* gene was significantly increased after RP with DMOG in all time points compared to controls (Figure 5e, OGD, *n* = 7; OGD + reperfusion, *n* = 4; OGD + reperfusion + DMOG, *n* = 4). Across all time points, DMOG administration significantly increased the expression compared to RP without DMOG.

## 4 DISCUSSION

### 4.1 CDCs as a model for I–R injury

In vivo models are typically used in preclinical studies to investigate myocardial I–R injury. Usually, the LCA of an experimental animal is occluded by ligatures, which interrupts blood flow. The vessel can then be reopened for reperfusion, if required (Totzeck et al., 2016). Although animal models do not exactly correspond to human physiology, they provide a useful approximation for investigating the interaction between different cell types as well as the influence of I–R injury, inflammatory processes, and the cardiac conduction system (Lindsey et al., 2018). On the other hand, investigating a pure in vitro cardiomyocyte culture represents the most reduced model. While the advantages lie in the relatively straightforward feasibility and isolated analysis of the effects of ischaemia and reperfusion on cardiomyocytes, the limitations of this restricted model are apparent. Due to the presence of other cell types, factors, and physiological circumstances, it is not possible to simply translate the findings into an in vivo situation. A model that combines the strengths of in vivo and in vitro approaches is the use of CDCs as a heterogeneous primary cell culture to analyse I–R injury in vitro. Although CDCs have primarily been considered a source of stem cells (Davis et al., 2010), they can differentiate into three cell types that are particularly relevant to the heart: cardiomyocytes, smooth muscle cells and endothelial cells (Carr et al., 2011). Thus, a valid model for studying I–R in CDCs would be able to identify possible therapeutic approaches under easily modifiable conditions without neglecting the (paracrine) influence of other important cell types apart from cardiomyocytes.

The CDCs on which this work was based were extracted from the hearts of neonatal mice and cultivated after isolation from the cardiosphere. Immunocytochemical analysis detected cardiomyocytes, smooth muscle cells, endothelial cells and stem cells by immunofluorescence (Figure 1b). Quantitative cell count analysis revealed that cardiomyocytes constituted the largest proportion of cells (34.3%), followed by smooth muscle cells (33.5%), endothelial cells (6.0%) and stem cells (5.8%) (Figure 1c). To create an in vitro model that more closely resembles a human heart than an isolated cardiomyocyte culture, it is important to consider the cell composition in relation to that of an adult human heart. Litviňuková *et al.* investigated 14 human donor hearts using single‐cell and single‐cell nuclear RNA sequencing, revealing regional differences (Litvinukova et al., 2020). While the composition of the CDC culture established in this study at passage 5 is not completely identical to that of the adult heart, it is a close approximation. Although the relative proportion of cardiomyocytes (30.1%) corresponds more to the atrium, the production of smooth muscle cells (21.2%) and endothelial cells (7.8%) approaches that of the ventricles. The relatively high proportion of stem cells in CDC culture has no analogy in adult humans, where a stem cell proportion of 0.01% is assumed (Leong et al., 2017).

Using primary cells from newborn mice to investigate I–R injury may seem unusual at first, but it is particularly relevant in the clinical setting for older populations (Beller et al., 2020). However, despite being more resilient to hypoxia (Ostadalova et al., 1998; Riva & Hearse, 1993), neonatal cardiomyocytes are often used in in vitro primary cell studies because of their simpler culture conditions (Date et al., 2002; Negoro et al., 2001; Tanaka et al., 1994). Another advantage of neonatal cardiomyocytes is that in contrast to adult cells, they can be used to study cell–cell contacts (Lindsey et al., 2018). However, several limitations of the CDC model should be acknowledged when interpreting these findings. First, the study used neonatal mouse CDCs, which may not fully reflect the biology of the adult myocardium, and the cultures are inherently heterogeneous, with approximately 20% of cells lacking detectable lineage markers. Similar to other stem‐ or progenitor‐derived cardiac models, CDCs do not achieve a fully ‘adult’ phenotype, and their metabolic and stress response profiles likely reflect a more immature state. The intrinsic heterogeneity may influence the stress responses observed. Distinct CDC subpopulations differ in their metabolic status, differentiation state and hypoxia‐response capacity, which may modulate susceptibility to reperfusion injury and cellular response to DMOG‐induced chemical hypoxia. For example, progenitor‐enriched subsets may exhibit greater resistance to oxidative stress, whereas more fibroblast‐like cells may display differential activation of the HIF pathway. Additionally, CDCs can be maintained only for a finite period before culture‐related drift occurs; therefore, the experiments in this study were performed without subcultivation of cardiospheres to minimize phenotypic degradation. With respect to the I–R simulation, reperfusion occurred rapidly as the medium lacking glucose was removed and immediately replaced with glucose‐containing medium, and the culture dish was transferred from hypoxia to ambient oxygen within seconds. Although this is faster and more controlled than clinical reperfusion during coronary intervention, it reproduces the essential shift in oxygen and nutrient availability that drives early reperfusion injury. Importantly, CDCs offer several advantages over 3D cardiospheres in I–R studies. As 2D heterogeneous monolayers, CDCs are more uniformly exposed to hypoxic and reperfusion conditions, easier to maintain and image, and allow single‐cell level assessment of injury pathways. Features that are more difficult to achieve in densely packed spherical aggregates with oxygen and nutrient gradients. Despite these limitations, these properties make CDCs a valuable and practical model for mechanistic studies of MI and for probing cellular responses to ischaemia–reperfusion in a controlled, reproducible manner.

### 4.2 PHI treatment

PHD inhibitors show promise in protecting and repairing the heart after ischaemic injury. The accumulation of HIF, which can be achieved through preconditioning, genetically reducing PHD activity or using pharmacological PHD inhibitors, has been associated with reduced infarct size and improved cardiac function in MI models. Furthermore, stabilization of HIF via hypoxia or PHI treatment enhanced heart regeneration in animal studies (Schreiber et al., 2019). These findings suggest new therapeutic approaches to treating ischaemic heart disease (IHD) and promoting cardiac tissue regeneration. Yet almost all experimental studies administered PHIs before or at the onset of ischaemia. Only four studies have demonstrated beneficial effects even when treatment is initiated after MI (Bao et al., 2010; Jatho et al., 2021; Nwogu et al., 2001; Philipp et al., 2006). However, all of these studies used permanent LCA ligation models and did not evaluate the efficacy of PHIs in clinically relevant I–R settings.

To the best of our knowledge, this is the first study to administer a PHI during reperfusion, a scenario that might be used in the clinic.

As LDH is also of great importance in clinical practice compared with other laboratory chemistry methods, this analysis in an in vitro model is of particular interest. When examining the release of LDH from CDCs, we found that the amount of LDH increased with OGD duration (Figure 2a). After changing the medium and 1 h of RP, LDH still increased compared to that in the control group, indicating continuous cell death during reperfusion. Additionally, the LDH assay showed a reduced release of the enzyme after RP with DMOG compared to RP without DMOG, indicating a lower number of dead cells during RP with DMOG. There was no longer a change compared to the control; therefore, it can be concluded that DMOG prevented the observed in vitro reperfusion damage. Both Sonic Hedgehog (Shh) and PDGFA signalling have been implicated in the regulation of apoptosis and necroptosis, and both pathways exert well‐described pro‐survival effects by activating downstream signalling cascades that suppress apoptotic and necroptotic cell death and promote cellular resilience under stress conditions (Kalra et al., 2021; Ghaleh et al., 2020). Our data indicate that both Shh and Pdgfa signalling pathways may contribute to the modulation of cell fate under ischaemia–reperfusion conditions. The Shh pathway has been implicated in promoting cell survival by activating anti‐apoptotic mechanisms and limiting necroptotic cell death (Ghaleh et al., 2020). Specifically, Shh signalling can induce the expression of Bcl‐2 family proteins and enhance mitochondrial integrity, thereby reducing apoptosis while also modulating oxidative stress pathways that are central to necroptosis. In our experiments, although OGD alone did not significantly alter *Shh* expression, DMOG treatment during reperfusion led to an upregulation of *Shh* at 6 and 8 h, suggesting a potential cytoprotective effect during the reperfusion period. Similarly, Pdgfa, a key mediator of proliferation and survival signalling, has been reported to counteract apoptosis by activating the phosphoinositide 3‐kinase/Akt and mitogen‐activated protein kinase pathways and may indirectly attenuate necroptotic signalling by stabilizing cell survival pathways. In line with these findings, the observed DMOG‐mediated increases in *Pdgfa* expression at later reperfusion time points could underlie the modest protective effects detected in LDH assays, supporting a mechanistic basis for cytoprotection that complements biochemical readouts. Together, these data suggest that activation of Shh and PDGF signalling may help shift the balance from cell death toward survival during ischaemia–reperfusion stress, highlighting potential targets for enhancing cardiomyocyte resilience. The protective effect of DMOG in ischaemic models of in vitro ischaemia is already known; however, in these cases, PHI was also administered before ischaemia (Singh et al., 2020; Sridharan et al., 2007). However, the results of this study suggest that the addition of DMOG leads to a decrease in cell death when administered after an ischaemic event.

We also showed that HIF‐1α protein stabilization was elevated after RP with DMOG compared with RP without DMOG in CDCs. It is well known that DMOG also acts as a PHI in cardiomyocytes and stabilizes HIF‐1α (Mayorga et al., 2016; Xie et al., 2015). HIF‐1α was detected by western blotting in vivo (Figure 3c) and immunohistochemistry (Figure 3d) in both the remote area and the AAR. The increased detection of HIF‐1α after I–R in the remote area is consistent with the results of other studies that were able to detect HIF‐1α after induced MI also in the unaffected myocardium. Based on these data, 1 h of RP with DMOG is sufficient to stabilize HIF‐1α to an extent that might prove to be protective for the myocardium.

To compare CDCs and in vivo I–R injury, a number of genes with different regulatory properties were investigated. It has long been known that changes in gene expression occur after a MI (Hama et al., 1995; Ono et al., 1998). A significant increase in *Vegfa* expression occurs as early as 1 h after a MI (Li et al., 1996). In our study, no significant changes in *Vegfa* expression were observed in vivo after I–R (Figure 4a). This correlated with the expression pattern of CDCs after 1 h of OGD. At later time points, there was a tendency for increased expression, especially in RP with DMOG. VEGF plays an important role in early revascularization after MI and has been shown to reduce infarct size in an ovine reperfused MI model (Olea et al., 2013).

Although PHDs are ubiquitous in almost all cells, the distribution of individual isoforms differs from organ to organ. *Phd3* is strongly expressed in the heart and is a target gene of HIF‐1 (Pescador et al., 2005). Therefore, it is increasingly expressed in the heart under hypoxia (Willam et al., 2006) and serves as a feedback mechanism for HIF‐1 (del Peso et al., 2003; Minamishima et al., 2009). Here, we found significantly increased expression in vivo only in the remote area, but not in the AAR, although there is evidence of PHD3 at the protein level in ischaemic and non‐ischaemic hearts according to LCA ligature (Figure 4b, (Neckar et al., 2018). After RP with DMOG, we observed a marked decrease in expression compared to RP without DMOG. This may further augment HIF stabilization and be beneficial for the heart after MI. In the pathophysiology of MI, *Ctgf* ensures that migrating fibroblasts differentiate into myofibroblasts and persist locally (Szabo et al., 2014). While the process of scar formation lasts for weeks to months, *Ctgf* is acutely expressed after MI (Ohnishi et al., 1998). This was confirmed in our study after I–R in vivo, which showed significantly increased expression of AAR (see Figure 3; del Peso et al., 2003). An analogy to this can be found in the CDCs obtained only by OGD and subsequent RP, but not by OGD alone (Figure 5c). At later time points (6 and 8 h), DMOG seemed to facilitate expression even further. A possible explanation for the increased expression after RP might be the strain on the cytoskeleton (Muehlich et al., 2007). Dependence on oxygen content in the regulation of *Ctgf* in different cell types (Higgins et al., 2004; Kroening et al., 2010; Lee et al., 2009) was not observed in the CDCs. The regulation of Connexin 43, a product of the *Gja1* gene, is complex and partially unclear as a result of myocardial I–R injury (Basheer et al., 2018; Wang et al., 2019). We observed an increase in CDCs after I–R in vivo, but no changes were detected in the CDCs after OGD and RP without DMOG. A significant increase in *Gja1* expression was only observed after RP with DMOG. Since the protein products of the *Gja1* gene have a cardioprotective effect (Basheer et al., 2018), this might be beneficial after MI. DMOG had a similar effect on gene expression of *Adrb1*. It was significantly upregulated after RP with DMOG compared with RP without DMOG. It has already been shown that short ischaemic phases of 30–60 min led to a significant increase in *Adrb1* expression. However, this effect decreases with longer periods of ischaemia (Ihl‐Vahl et al., 1995). The fact that *Adrb1* expression in CDCs was significantly increased with DMOG compared to RP without DMOG suggests the involvement of the HIF signalling pathway. However, to date *Adrb1* is not known to be a HIF target gene. Nonetheless, it has been shown that the β‐adrenergic signalling pathway can delay the development of heart failure after MI (White et al., 2000).

The protective effect of PHI in cases of MI is well documented. To make progress in the clinical context, it is essential to stay close to real‐life situations, even in model experiments. As an approach to acute and post‐acute therapy, treatment can be initiated only as early as the diagnosis is confirmed or intervention is completed. Here, we demonstrated that CDCs serve as an effective model for I–R in vitro, which closely resembles the in vivo situation. Furthermore, we demonstrated for the first time that administering PHI during reperfusion reduces cell death and upregulates beneficial and protective genes, such as *Pdgfa* and *Gja1*. In a clinical setting, PHI can be administered immediately before or during recanalization or lysis therapy to achieve protective HIF stabilization and improve patient outcomes. However, this is a long way to go, and the next step would be to test this procedure in vivo.

## AUTHOR CONTRIBUTIONS

Timm Schreiber and Joachim Fandrey conceptualized and designed the study. Data acquisition was performed by Tristan Leu and Luca Salhöfer. UBHC supervised the in vivo surgery. Data analysis and interpretation was performed by Tristan Leu, Luca Salhöfer and Timm Schreiber. Tristan Leu and Timm Schreiber drafted the manuscript. All authors critically revised the manuscript, approved its final version and agree to be accountable for all aspects of the work in ensuring that questions related to the accuracy or integrity of any part of the work are appropriately investigated and resolved. All persons designated as authors qualify for authorship, and all those who qualify for authorship are listed.

## CONFLICT OF INTEREST

None declared.

## FUNDING INFORMATION

None.

## Data Availability

The data that support the findings of this study are available from the corresponding author upon reasonable request.

## 1

**Table:**

Comparison	*Adrb1*	*Ccnd2*	*Cdk2*	*Ctgf*	*Gja1*	*Ldha*	*Pdgfa*	*Phd2*	*Phd3*	*Shh*	*Vegf*	LDH
0 h OGD vs. 0 h OGD + 1 h DMOG	< 0.0001	< 0.0001	< 0.0001	< 0.0001	< 0.0001	> 0.9999	> 0.9999	< 0.0001	< 0.0001	< 0.0001	> 0.9999	> 0.9999
0 h OGD vs. 1 h OGD	0.9998	0.9997	> 0.9999	> 0.9999	> 0.9999	0.675	0.9893	> 0.9999	0.9695	0.9951	0.999	0.4731
0 h OGD vs. 1 h OGD + 1 h RP	> 0.9999	> 0.9999	> 0.9999	0.221	> 0.9999	0.5881	0.996	> 0.9999	0.9443	> 0.9999	0.3054	0.9989
0 h OGD vs. 1 h OGD + 1 h RP + DMOG	< 0.0001	< 0.0001	< 0.0001	< 0.0001	< 0.0001	> 0.9999	< 0.0001	< 0.0001	< 0.0001	< 0.0001	> 0.9999	> 0.9999
0 h OGD vs. 2 h OGD	> 0.9999	> 0.9999	> 0.9999	0.9907	> 0.9999	0.6147	0.6611	> 0.9999	0.9546	> 0.9999	0.9989	0.0789
0 h OGD vs. 2 h OGD + 1 h RP	> 0.9999	> 0.9999	>0.9999	0.4805	> 0.9999	0.6275	0.8011	0.9997	0.9546	> 0.9999	0.9988	0.998
0 h OGD vs. 2 h OGD + 1 RP + DMOG	< 0.0001	< 0.0001	< 0.0001	< 0.0001	< 0.0001	> 0.9999	< 0.0001	< 0.0001	< 0.0001	< 0.0001	> 0.9999	> 0.9999
0 h OGD vs. 4 h OGD	0.9999	0.8276	0.988	0.916	> 0.9999	0.7453	0.1106	> 0.9999	0.9546	0.9967	0.9997	0.0062
0 h OGD vs. 4 h OGD + 1 h RP	> 0.9999	0.9966	0.9927	> 0.9999	> 0.9999	0.6519	0.2741	0.9999	0.9546	0.9981	0.9984	0.9991
0 h OGD vs. 4 h OGD + 1 h RP + DMOG	< 0.0001	< 0.0001	< 0.0001	< 0.0001	< 0.0001	> 0.9999	< 0.0001	< 0.0001	< 0.0001	< 0.0001	> 0.9999	> 0.9999
0 h OGD vs. 6 h OGD	0.9965	0.9908	0.4962	0.8836	> 0.9999	0.6275	0.0351	> 0.9999	0.9695	0.9998	0.6419	0.0158
0 h OGD vs. 6 h + 1 h RP	> 0.9999	0.9969	0.9933	> 0.9999	> 0.9999	0.6275	0.0925	0.9997	0.9443	0.9982	0.9989	> 0.9999
0 h OGD vs. 6 h OGD + 1 h RP + DMOG	< 0.0001	< 0.0001	< 0.0001	< 0.0001	< 0.0001	> 0.9999	< 0.0001	< 0.0001	< 0.0001	< 0.0001	> 0.9999	> 0.9999
0 h OGD vs. 8 h OGD	0.9816	0.7975	0.0748	0.8979	0.9992	0.6399	0.0033	0.9996	0.9695	0.9618	0.9989	0.0002
0 h OGD vs. 8 h OGD + 1 h RP	> 0.9999	> 0.9999	0.6694	> 0.9999	> 0.9999	0.6399	0.0925	0.9996	0.9546	0.9982	0.9989	0.9714
0 h OGD vs. 8 h OGD + 1 h RP + DMOG	< 0.0001	< 0.0001	< 0.0001	< 0.0001	< 0.0001	> 0.9999	< 0.0001	< 0.0001	< 0.0001	> 0.9999	> 0.9999	> 0.9999
1 h OGD vs. 1 h OGD + 1 h RP	> 0.9999	0.9378	0.983	0.0086	> 0.9999	> 0.9999	> 0.9999	> 0.9999	> 0.9999	0.842	0.9393	0.9856
1 h OGD vs. 1 h OGD + 1 h RP + DMOG	< 0.0001	< 0.0001	< 0.0001	< 0.0001	< 0.0001	> 0.9999	< 0.0001	< 0.0001	> 0.9999	> 0.9999	> 0.9999	0.5061
1 h OGD + 1 h RP vs. 1 h OGD + 1 h RP + DMOG	< 0.0001	> 0.9999	< 0.0001	< 0.0001	< 0.0001	> 0.9999	< 0.0001	> 0.9999	> 0.9999	< 0.0001	> 0.9999	0.9994
2 h OGD vs. 2 h OGD + 1 h RP	> 0.9999	> 0.9999	> 0.9999	0.0086	> 0.9999	> 0.9999	> 0.9999	>0.9999	> 0.9999	> 0.9999	> 0.9999	0.6156
2 h OGD vs. 2 h OGD + 1 RP + DMOG	< 0.0001	< 0.0001	< 0.0001	< 0.0001	< 0.0001	> 0.9999	< 0.0001	< 0.0001	> 0.9999	< 0.0001	> 0.9999	0.0836
2 h OGD + 1 h RP vs. 2 h OGD + 1 RP + DMOG	< 0.0001	> 0.9999	< 0.0001	< 0.0001	> 0.9999	> 0.9999	< 0.0001	> 0.9999	> 0.9999	< 0.0001	< 0.0001	0.9984
4 h OGD vs. 4 h OGD + 1 h RP	> 0.9999	> 0.9999	> 0.9999	0.3677	0.9994	> 0.9999	> 0.9999	> 0.9999	> 0.9999	> 0.9999	> 0.9999	0.0962
4 h OGD vs. 4 h OGD + 1 h RP + DMOG	< 0.0001	< 0.0001	> 0.9999	< 0.0001	< 0.0001	> 0.9999	< 0.0001	< 0.0001	> 0.9999	< 0.0001	> 0.9999	0.0087
4 h OGD + 1 h RP vs. 4 h OGD + 1 h RP + DMOG	< 0.0001	< 0.0001	> 0.9999	> 0.9999	> 0.9999	> 0.9999	< 0.0001	< 0.0001	> 0.9999	< 0.0001	> 0.9999	0.9998
6 h OGD vs. 6 h + 1 h RP	> 0.9999	> 0.9999	0.9998	0.3086	> 0.9999	> 0.9999	> 0.9999	> 0.9999	> 0.9999	> 0.9999	0.9988	0.2379
6 h OGD vs. 6 h OGD + 1 h RP + DMOG	< 0.0001	< 0.0001	< 0.0001	< 0.0001	< 0.0001	> 0.9999	< 0.0001	< 0.0001	> 0.9999	< 0.0001	< 0.0001	0.031
6 h OGD + 1 h RP vs. 6 h OGD + 1 h RP + DMOG	< 0.0001	< 0.0001	> 0.9999	< 0.0001	< 0.0001	> 0.9999	< 0.0001	< 0.0001	> 0.9999	< 0.0001	< 0.0001	> 0.9999
8 h OGD vs. 8 h OGD + 1 h RP	0.9968	0.9917	0.9998	0.891	0.9991	> 0.9999	> 0.9999	> 0.9999	> 0.9999	> 0.9999	> 0.9999	0.013
8 h OGD vs. 8 h OGD + 1 h RP + DMOG	< 0.0001	< 0.0001	< 0.0001	< 0.0001	< 0.0001	> 0.9999	< 0.0001	< 0.0001	< 0.0001	< 0.0001	< 0.0001	0.0003
8 h OGD + 1 h RP vs. 8 h OGD + 1 h RP + DMOG	< 0.0001	< 0.0001	< 0.0001	< 0.0001	< 0.0001	> 0.9999	< 0.0001	< 0.0001	< 0.0001	< 0.0001	< 0.0001	0.9888
OGD vs. OGD + 1 h RP	0.143	0.2083	0.334	< 0.0001	0.6735	0.837	0.2251	0.1162	0.6577	0.0223	0.8546	< 0.0001
OGD vs. OGD + 1 h RP + DMOG	0.1656	0.2066	0.8977	< 0.0001	0.0242	0.2279	0.4576	0.9046	0.024	0.0731	0.1519	< 0.0001
OGD + 1 h RP vs. OGD + 1 h RP + DMOG	0.004	> 0.9999	0.2264	0.9759	0.0068	0.0727	0.0398	0.1977	0.0013	0.899	0.3862	0.0271

The table summarizes the results of a two‐way ANOVA assessing the effects of time and treatment, as well as their interaction, on the expression levels of multiple genes and on LDH release. Reported values represent the statistical significance for main effects and interaction terms for each measured parameter. Experimental replicates: OGD, *n* = 3–7; OGD + reperfusion, *n* = 3–4; OGD + reperfusion + DMOG, *n* = 3–4.
